# Contribution of the Axon Initial Segment to Action Potentials Recorded Extracellularly

**DOI:** 10.1523/ENEURO.0068-18.2018

**Published:** 2018-06-05

**Authors:** Maria Teleńczuk, Romain Brette, Alain Destexhe, Bartosz Teleńczuk

**Affiliations:** 1 Laboratory of Computational Neuroscience, Unité de Neurosciences, Information et Complexité, CNRS, Paris 91190 Gif sur Yvette, France; 2 Institut National de la Santé et de la Recherche Médicale, CNRS, Institut De La Vision, Sorbonne Universités, Paris F-75012, France

**Keywords:** axon initial segment, computational modelling, extracellular action potential

## Abstract

Action potentials (APs) are electric phenomena that are recorded both intracellularly and extracellularly. APs are usually initiated in the short segment of the axon called the axon initial segment (AIS). It was recently proposed that at the onset of an AP the soma and the AIS form a dipole. We study the extracellular signature [the extracellular AP (EAP)] generated by such a dipole. First, we demonstrate the formation of the dipole and its extracellular signature in detailed morphological models of a reconstructed pyramidal neuron. Then, we study the EAP waveform and its spatial dependence in models with axonal AP initiation and contrast it with the EAP obtained in models with somatic AP initiation. We show that in the models with axonal AP initiation the dipole forms between somatodendritic compartments and the AIS, and not between soma and dendrites as in the classical models. The soma–dendrites dipole is present only in models with somatic AP initiation. Our study has consequences for interpreting extracellular recordings of single-neuron activity and determining electrophysiological neuron types, but also for better understanding the origins of the high-frequency macroscopic extracellular potentials recorded in the brain.

## Significance Statement

Action potentials in most neurons initiate in the axon initial segment (AIS). However, the AIS is often neglected in computational studies. We studied the consequences of this initiation mechanism on the extracellular signatures of action potentials. We show that at the time of AP initiation AIS forms a dipole with the soma. The peak-to-peak amplitude of the extracellular action potential (EAP) generated by this dipole changes little with changing soma–AIS distance, while the width of the EAP increases with the soma–AIS distance. This may help to monitor dynamic changes in the soma–AIS distance in experimental *in vivo* recordings.

## Introduction

Action potentials (APs) are the main output of neuronal computation arising due to neuronal membrane excitability. The most direct method to detect APs is by intracellular recordings for which a glass pipette is inserted into the soma. However, the sample size of neurons recorded with this technique is limited. Another method of AP detection uses extracellular electrodes whose densities can be greatly increased thanks to silicon technology opening the possibility of massive recordings from large samples of neurons (Stevenson and Kording, 2011; Jun et al., 2017). The drawback of this method is that the discrimination of separate neurons and their types based on extracellular recordings is not trivial (Barthó et al., 2004) and requires a detailed model of how the extracellular signature of the APs is generated.

APs also contribute to the local field potentials (LFPs) and electroencephalograms (EEGs) recorded far from the neuronal source. In particular, the high-frequency components of these signals can relate to the firing rates of large population of neurons (Reimann et al., 2013). High-frequency LFP is also known to be sensitive to the neuronal responses at single-neuron and single-trial levels (Teleńczuk et al., 2015). Therefore, APs can be as important as the passive dendritic and synaptic currents for understanding the LFP or EEG and in particular their high-frequency components.

The extracellular signature of APs has been a topic of computational studies (Bédard et al., 2004; Gold et al., 2006; Milstein and Koch, 2008). These studies emphasize the role of passive currents and dendritic compartments in the generation of the action potentials. However, in most of those models APs were initiated in the soma. It is now well established that the AP often initiates in the axon initial segment (AIS; Stuart et al., 1997a,b), which gives a characteristic kink at the AP onset when recorded somatically (Naundorf et al., 2007). This kink can be explained by the “critical resistive coupling model,” according to which the AP is initiated through the strong resistive coupling between a small AIS and a large soma (Brette, 2013; Telenczuk et al., 2017). In this mechanism of AP initiation, AIS and soma form effectively a current dipole.

We studied the contribution of the soma–AIS dipole to the extracellular field and its effect on the shape and peak-to-peak amplitude of the extracellular AP (EAP). In particular, we studied the EAP from realistic model neurons with AIS-based initiation and compared it with models for which the sodium channel density was modified to initiate the AP somatically. By means of computational modeling, we show that the AIS contributes significantly to the EAP. Although the localization and length of the AIS have only a minor effect on the appearance of the AP recorded intracellularly from the soma, the presence of AIS has a large impact on the shape of the EAP.

We believe that these findings improve our understanding of the close-field and far-field contribution of the AP to the extracellular potentials in the brain. It will also help to interpret recordings of various signals ranging from the EAP, through LFP to EEG.

## Materials and Methods

### Detailed morphology model

We used a detailed morphology model (physiological Na_v_ model) of the rat neocortex, layer 5 pyramidal neuron described in the study by Hallermann et al. (2012), whose morphology and ion channels are modeled so as to give good fit to the experimental data. Most importantly, in this model action potentials initiate in the AIS, as is the case in real neurons. The details of the model can be found in the study by Hallermann et al. (2012). The channel kinetics are the same as in the original model, which contained two types of sodium channels (putative Na_v_1.2 and Na_v_1.6). Na_v_1.2 channels were placed in both the soma and axon, and the kinetics matched experimental recordings. The Na_v_1.6 channel was present only in the axon; its activation curve was shifted by 2 mV toward a more negative potential to account for the lower threshold of AP initiation in the AIS.

The density of the sodium channels in the soma was 500 pS/μm^2^, while in the AIS it varied between 1452 and 8392 pS/μm^2^ (see Fig. 2C, physiological Na_v_ model). To compare the results of the original model to the neuron where the action potential initiates in the soma, we reduced the density of the sodium channels in the AIS just below that in the soma to 480 pS/μm^2^ throughout the length of the initial segment of the axon (70 μm; see Fig. 2C, reduced Na_v_ model). The density of the sodium channels in the soma remained the same as in the original model (500 pS/μm^2^). This was enough for an action potential to initiate in the soma. We note that there are fewer sodium channels in the altered model, leading to lower transmembrane current intensities; therefore, comparison of the absolute peak-to-peak amplitudes of the extracellular potentials is not possible. In Figures 5 and 6, we normalized the potentials to the highest absolute value of the potential. To trigger the action potential in either model, we injected current steps (1.5 nA; duration, 15 ms) to the soma.

In the detailed morphology model, in all the calculations, the soma is modeled as a cylinder. However, in the figures we represent it as a triangular shape for easier visualization of the morphology of the cell.

### Soma–axon model

We used a simple neuron consisting of a soma (20 × 30 μm, 6 segments) and an axon (1 × 50 μm, 10 segments) available on ModelDB (access number: 135839), described by Yu et al. (2008). See Figure 8*A* for sample schematics of the shape of the neuron. The simulation was controlled from Python using the Neuron–Python interface (Hines et al., 2009).

### Linear source approximation

To estimate the extracellular potentials we used the linear source approximation (LSA) method, which calculates the summed potential generated by currents originating from line sources with known sizes and positions. This method is known to be more precise than approximating the currents by point sink and sources (Holt, 1997; Wilson and Bower, 1992). We then applied the LSA estimation to cylinders obtained from the segmentation by neuron simulator (Hines and Carnevale, 1997). The field was calculated using the LSA implementation of NeuronEAP Python library (Telenczuk and Telenczuk, 2016). In all calculations, we used an extracellular conductivity of 0.3 Sm (Nunez and Srinivasan, 2006).

In Figure 8, we removed the baseline from the extracellular potential by calculating an average potential in a window from 2 to 1 ms before the peak of the action potential.

### Code accessibility

The code for the figures of the full morphology model is deposited and available at Zenodo (DOI: 10.5281/zenodo. 1222159). We used the full morphology model published by Hallermann et al. (2012) and the simplified model published by Yu et al. (2008) in their original form and with altered parameters, as stated where appropriate. To calculate the local field potential, we used the NeuronEAP Python library (Telenczuk and Telenczuk, 2016).

## Results

### AP is initiated in the AIS and gives a characteristic “kink” to the somatic potential

**Figure 1.**
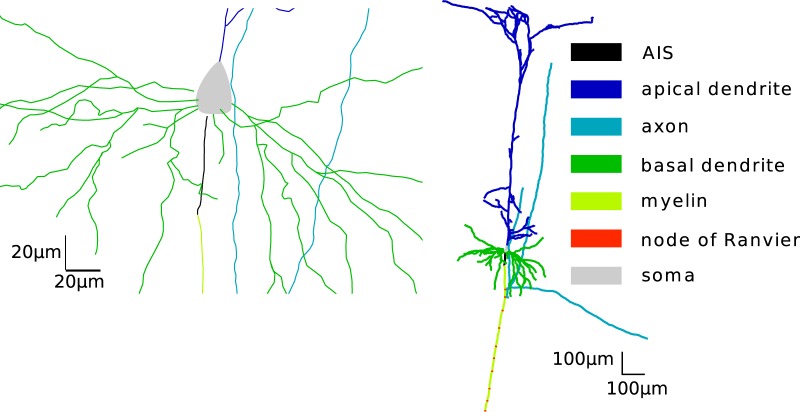
Morphology of the full compartmental model. Left, Zoom into the AIS.

**Figure 2.**
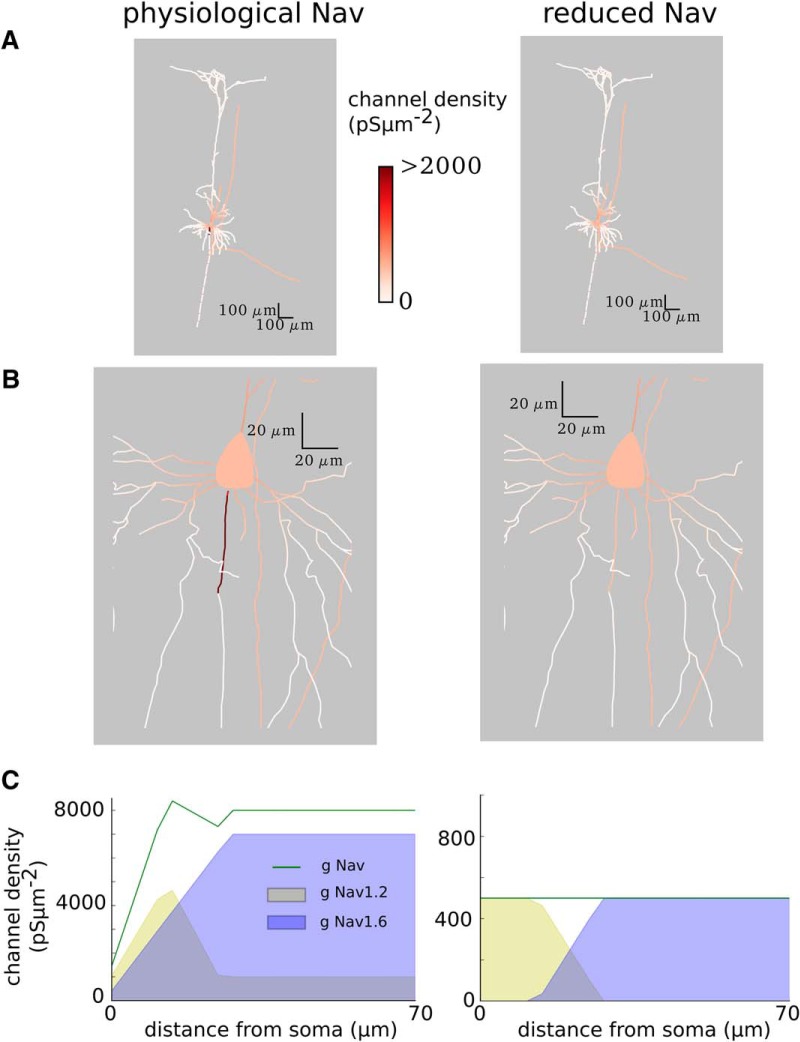
Sodium distribution within the neuron. Color scale shows the channel conductance per membrane area. Left, Physiological Na_v_ model. Right, Reduced Na_v_ model. ***A***, Full morphology. ***B***, Zoom into the soma and the initial segments of the axon. ***C***, Concentrations of two different types of sodium channels (Na_v_1.2 and Na_v_1.6) in the AIS (at 0 μm, AIS is attached to the soma; 69.90 μm is its far end). Note that in both models, the density of Na_v_1.2 channels in the soma is 500 pS/μm^2^, while there are no Na_v_1.6 channels.

To determine the contribution of an AP to the extracellular potentials recorded around the neuron, we performed simulations of a detailed reconstruction of a thick-tufted pyramidal neuron (neocortex, layer 5, rat; Fig. 1). The morphology reflected real reconstructed neurons with all neuronal compartments, including an axon and dendrites. The densities and the kinetics of sodium (Na) and potassium (K) channels in soma and axon were constrained by the experimental data. In particular, two different types of sodium channels were introduced (referred to as Na_v_1.2 and Na_v_1.6; see Materials and Methods) with different voltage activation threshold and different distribution of the channel density across the axosomatic axis (Fig. 2, left). Overall, this model has been found to match well the properties of AP initiation in cortical neurons (Hallermann et al., 2012; Telenczuk et al., 2017).

**Figure 3.**
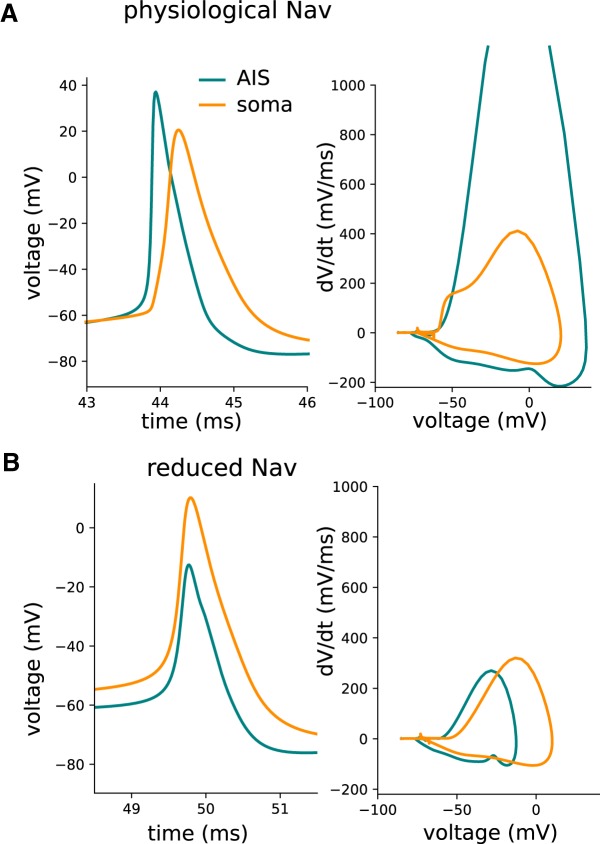
Action potentials in two different locations: soma (orange) and distal end of AIS (blue). The AP is shown both in the time domain (left) and in a phase-plot (right). ***A***, Physiological Na_v_ model. ***B***, Reduced Na_v_ model.

Importantly, in this model the action potential initiates distally from the soma, in the AIS, and later triggers a somatic AP, which is in agreement with physiological recordings (Stuart et al., 1997a). This mechanism of AP initiation gives a characteristic “kink” at the onset of the somatic AP (Fig. 3*A*). This is consistent with resistive coupling between the AIS and soma (Telenczuk et al., 2017). The resistive coupling model predicts that the soma and AIS form a dipole at AP initiation, which should be observed in the extracellular potential.

### AIS generates positive peak at the onset of the EAP

We first characterized the waveform of the EAP. Previous models displaying somatic AP initiation have indicated that mainly sodium currents in the soma and dendrites might contribute to the initial phases of the EAP, whereas later phases are shaped by the repolarization mediated by potassium currents in these compartments (Gold et al., 2006). In contrast, in these models axon, distal dendrites and the capacitive current contribute little to the EAP.

**Figure 4.**
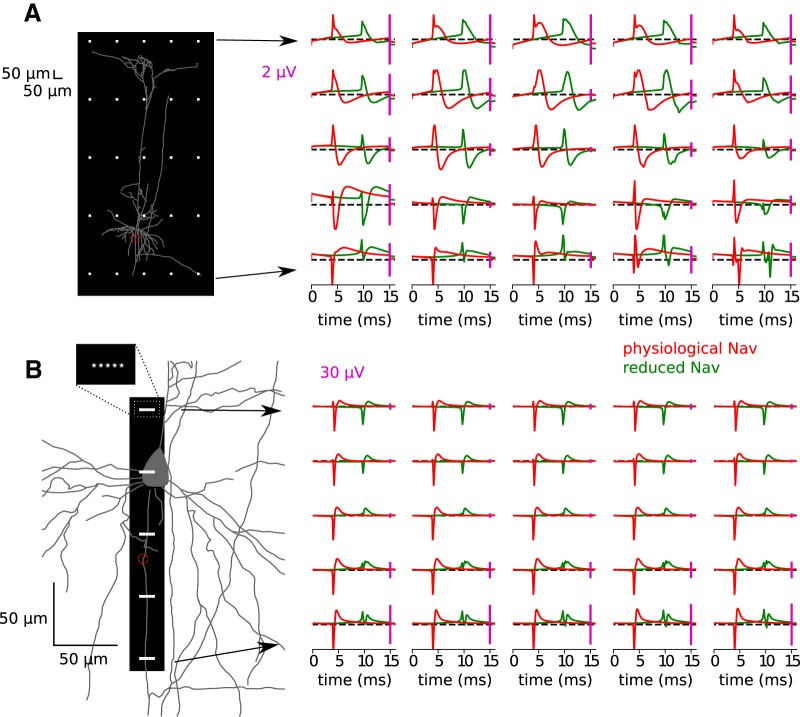
Extracellular potentials (right) measured at different locations (white dots within the black rectangle, left) for the physiological Na_v_ model (red) and reduced Na_v_ model (green). Calibrations: ***A***, 2 μV; ***B***, 30 μV. The *y*-scale is adjusted in each panel separately for better visualization of the EAPs. ***A***, Full morphology. ***B***, Zoom in to the soma and initial part of the axon. The distal end of the AIS is marked with a red circle (***A***, ***B***).

We re-evaluated the contribution of the AP to the extracellular potentials in the more realistic model with AIS-initiated AP. First, we calculated and plotted the EAP recorded in the perisomatic area covering soma, proximal dendrites, and the AIS in the physiological Na_v_ model (Fig. 4). Consistently with previous results (Gold et al., 2006), we found a large and sharp negative peak, due to sodium inflow, followed by a broad positive peak, due to potassium-based repolarization of the soma and dendrites. Interestingly, in some electrodes (around and above soma) these peaks were preceded by a sharp positive deflection reflecting strong axial currents flowing between AIS and soma at the onset of the AP.

To confirm that this initial positive peak is related to the resistive coupling between soma and AIS forming a dipole, we lowered the densities of sodium channels in the AIS (reduced Na_v_ model; Fig. 2, right). As expected, this modification led to the somatic initiation of the AP, which appears simultaneously at soma and AIS (these two compartments being almost isopotential), and longer AP latency due to higher threshold (Fig. 3). The EAP waveforms obtained in this modified model lack the initial positivity consistently with the results of Gold et al. (2006). In addition, in the reduced Na_v_ model the maximum of the EAP decreases more sharply with distance, especially in the area occupied by dendrites (Fig. 4*A*, middle row). We emphasize, though, that such a model is inconsistent with the experimental observations of AP initiation, which support axonal (AIS) rather than somatic initiation of APs.

### The AIS enhances the peak-to-peak amplitude at broad spatial ranges

**Figure 5.**
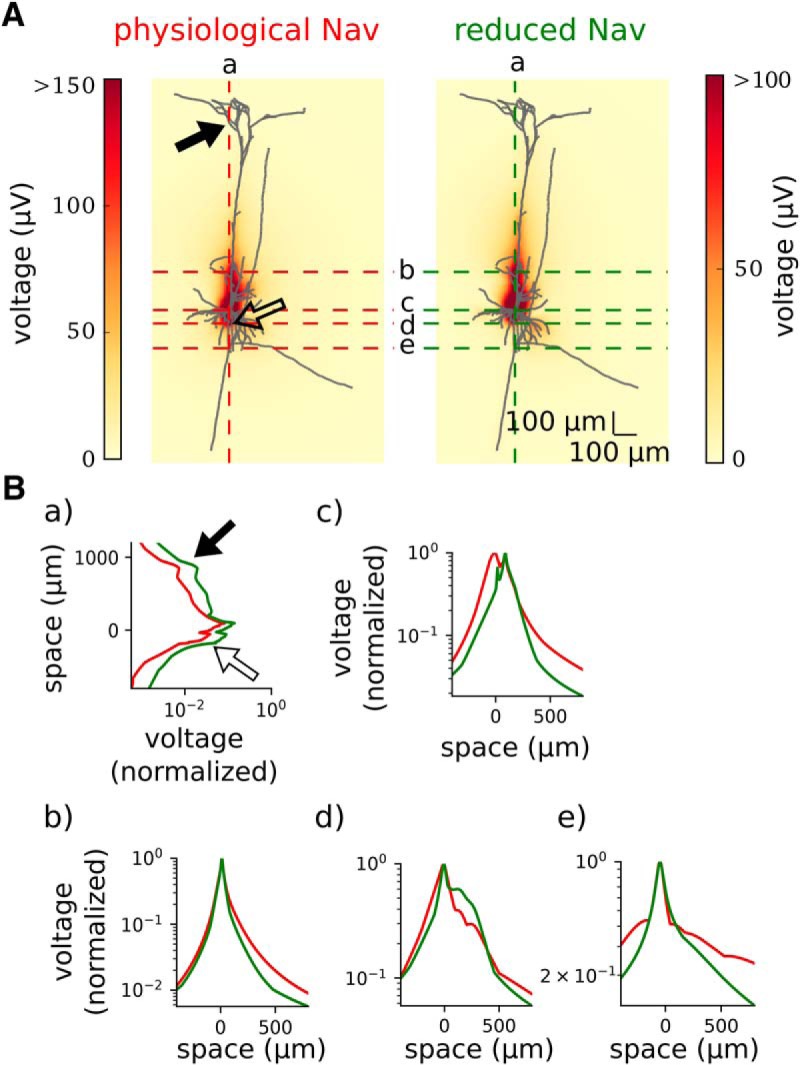
Maximum peak-to-peak amplitude of the EAP calculated in the different places of the field. ***A***, Full morphology imposed on the maximum peak-to-peak amplitude (heatmap; colorbar on the right) in the physiological Na_v_ model (left) and reduced Na_v_ model (right). The EAPs with the highest peak-to-peak amplitudes are obtained in the somatic region of the neuron (dark red color in heat map; see also Fig. 6 for a zoom-in). ***B***, Dotted lines show the axes along which subpanels ***a–e*** are calculated. Soma is centered at the position (0 μm, 0 μm). ***B***, Maximum peak-to-peak potential normalized to the largest value of the potential for each model separately: the physiological Na_v_ model (red) and reduced Na_v_ model (green). The potential is given in the logarithmic scale. ***a***, Signal recorded in the vertical axis passing through the soma. ***b***, Signal recorded in the horizontal axis passing 200 μm above the soma. ***c***, Signal recorded in the horizontal axis passing through the soma. ***d***, Signal recorded in the horizontal axis passing through the AIS. ***e***, Signal recorded in the horizontal axis passing through 200 μm below the soma. Arrows point to the area occupied by a dendrite, which creates a sharp peak in the measured extracellular potential.

**Figure 6.**
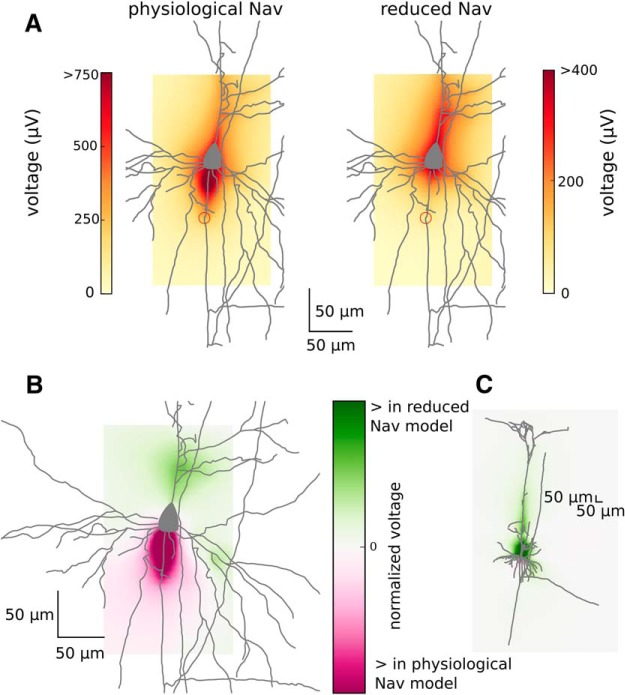
Comparison of peak-to-peak amplitude in physiological and reduced Na_v_ models. ***A***, Zoom-in to the maximum peak-to-peak amplitude of the EAP (shown as a heatmap; colorbar is on the right-hand side) generated by the physiological Na_v_ model (left) and reduced Na_v_ model (right). The peak-to-peak amplitude around AIS (red circles, distal end) is higher in the model with axonal initiation (physiological Na_v_ model). ***B***, ***C***, Difference between normalized peak-to-peak amplitudes (heatmap; colorbar on the right) of the EAP obtained from physiological and reduced Na_v_ models: the zoomed-in view (***B***) and full morphology (***C***).

The peak-to-peak amplitude decays with the distance from the neuron (Fig. 5). It is highest around soma and AIS, where the largest inflow of sodium and outflow of potassium during the AP takes place. Lowering sodium channel density such that AP initiated somatically attenuates the peak-to-peak amplitude of the EAP, which is expected from the decrease of the total membrane current in the low-sodium model (data not shown). Importantly, the reduction of peak-to-peak amplitude was most pronounced in the axonal region, especially in the proximity of the axon segment previously acting as the AIS (Fig. 6).

Next, we plotted the peak-to-peak amplitude of the EAP across four lines perpendicular to the somatodendritic axis (Fig. 5). Close to the neuron, the profile of the peak-to-peak amplitude was nonmonotonic due to the complex morphology of the neuron, but it monotonically decreased with distance further away from the source. Again, due to the larger total membrane current the peak-to-peak amplitude is greater in standard sodium models compared with the low-sodium modification across all distances.

### AIS contribution to EAP can be approximated by a soma–AIS dipole

**Figure 7.**
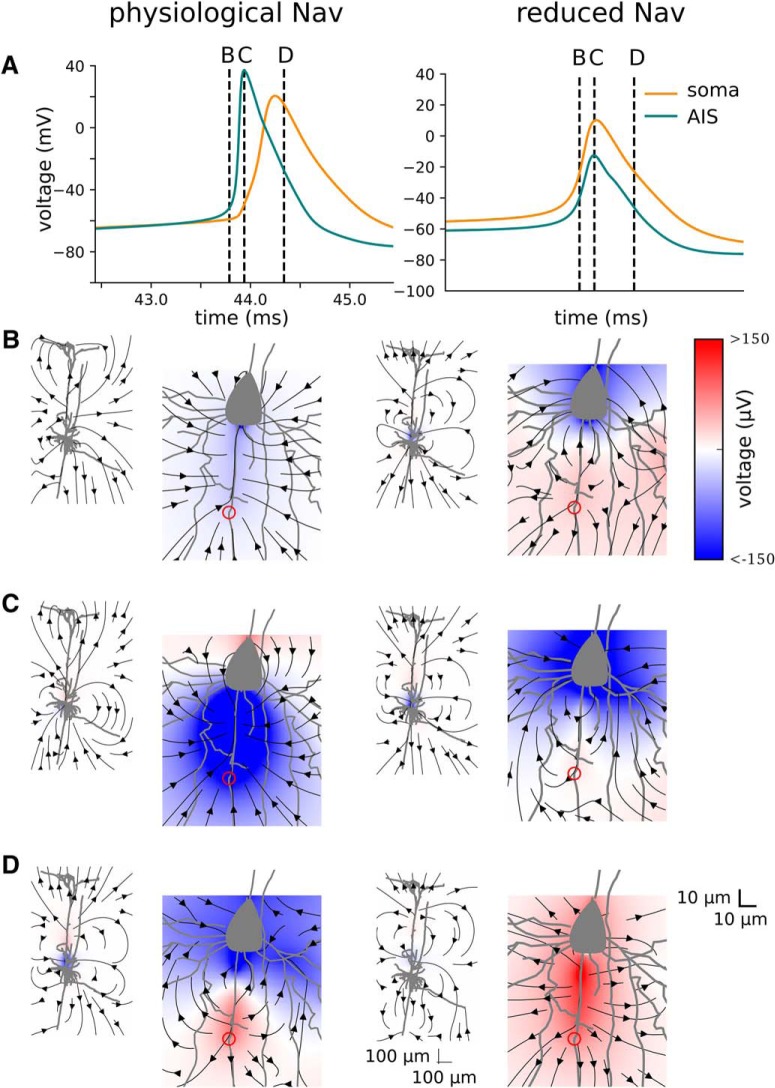
EAP at different time points in the physiological Na_v_ model (left) and reduced Na_v_ model (right). ***A***, Intracellular APs in the soma (orange) and in the end of the AIS (blue). Dotted vertical lines show at which time points ***B–D*** are recorded. ***B–D***, Extracellular potentials (colormap; see the colorbar on the right, red is positive and blue is negative) and electrical current (arrows) at different times of APs plotted for around the whole morphology (left) and around the soma–AIS region (right). ***B–D***, Recordings were made as follows: at 0.15 ms before the peak of the AP in the AIS (***B***), at the peak of the AP in the AIS (***C***), and 0.4 ms after the peak of the AP in the AIS (***D***). In the physiological Na_v_ model, the AP initiates in the AIS (red circles) giving rise to a dipolar potential (AIS negative, soma positive; ***C***, left), which later reverses in polarity (AIS positive, soma negative; ***D***, left). In contrast, the reduced Na_v_ model produces a large dipole that encompasses the axon, soma, and proximal dendrites (soma–dendrites dipole).

In the physiological Nav model, at the moment of AP initiation the axial current and the extracellular currents form a current loop. This current loop produces extracellular potentials with dipolar configuration i.e. negative potential around AIS (sink) and positive potential around soma and proximal dendrites (source); Fig. 7*B*. This relation is reversed during the repolarization phase of the AP, during which the polarities of AIS and somatodendritic compartments are reversed (Fig. 7*C*). Such a configuration of sinks and sources will be referred to as the soma–AIS dipole. In the model with somatic AP initiation (reduced Na_v_ model), the soma and AIS are almost isopotential, so no current flows between them. In this case, the soma–AIS dipole is not formed, but it is replaced by the sink in the soma (or source after the inversion) and the source in the proximal dendritic tree (soma–dendrites dipole)

The extracellular potentials obtained from the detailed morphologic models contain a mixture of contributions from passive dendritic compartments and active axonal/somatic compartments, giving rise to a complex configuration of current sinks and sources. To isolate the effects of the soma–AIS dipole and its contribution to the far-field potential, we decided to further corroborate the consequences of the “critical resistive coupling” with a simplified electric dipole model. We reduced the model to a cylindrical soma and an axon. All Na_v_ and K channels were placed in the AIS modeled as a 5-μm-long segment of the axon located 45 μm distally from the soma. As shown previously (Telenczuk et al., 2017), this model approximates well the dipolar field that was also observed in the detailed morphologic model described above (Fig. 7).

**Figure 8.**
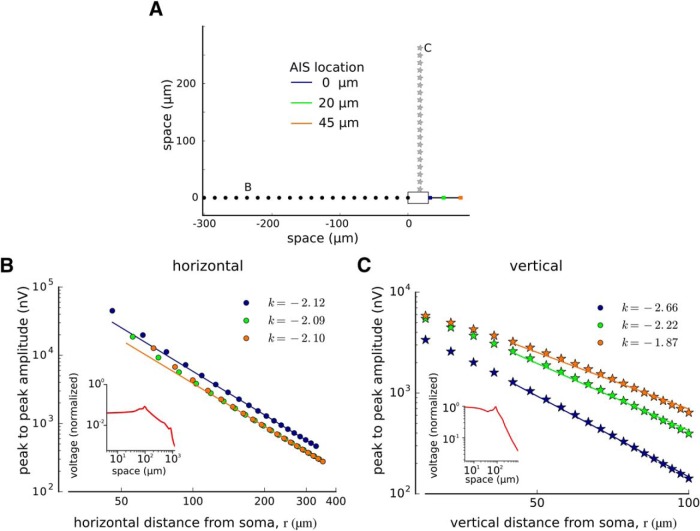
Extracellular potentials calculated from the soma–axon model with the AIS at three different distances: 0 μm from the soma (blue), 20 μm from the soma (green), and 45 μm from the soma (orange). ***A***, Calculation of EAP amplitude in a simplified soma–AIS model. The drawing represents the cell body (white rectangle) and the axon (black) with the AIS at different locations (color coded). The location of the measurement points along horizontal (black dots) and vertical axes (gray stars). ***B***, ***C***, Peak-to-peak amplitude of the EAP along the soma–AIS axis (***B***, black dots in ***A*** show the recording locations) and vertically to the axon (***B***, gray stars in ***A***) in a double-logarithmic scale. Color lines correspond to different positions of the AIS (see color code in ***A***). Insets show example profiles of AIS amplitudes obtained in full morphologic model (compare Fig. 5*B*) along the respective axes. The decay of far-field potential with horizontal distance is well approximated with a power law, *r^k^*. The exponent, *k*, estimated from the slope of linear fit to the log-transformed potential, and the log distance is close to −2 along the horizontal axis (***B***, the value of *k* estimated for each model is given in the legend). The *k* exponent can be even smaller than −2 for vertical axis (***C***) due to the dependence of the extracellular potentials on the angle from the dipole axis (Fig. 9, θ).

We calculated the extracellular potential generated by this model neuron along the following two lines: the line that extended from the soma–AIS axis (Fig. 8*A*, dots) and the line that extended from the soma vertically to the axon (Fig. 8*A*, stars). We then repeated the calculation for three different positions of AIS (0, 20, and 45 μm away from the soma).

First, we analyzed horizontal measurements of the peak-to-peak amplitude EAP. They decayed monotonically with the distance from the soma (Fig. 8*B*). The absolute peak-to-peak amplitudes of EAP depended only slightly on the AIS position (Fig. 8*B*, color lines). To determine the law of peak-to-peak amplitude decay, we fitted a linear function in double logarithmic scale (i.e., both the peak-to-peak amplitude and recording distance, *r*, were log transformed). The slope of this function provided the estimate of the power law exponent (*k* in *r^k^* relation). We found that the exponent was similar for all soma–AIS distances (k≈−2). Next, we checked whether the same holds for vertical recordings, but we found instead that the exponent depended on the soma–AIS distance ranging from *k* = −2.66 to −1.87 (Fig. 8*C*).

**Figure 9.**
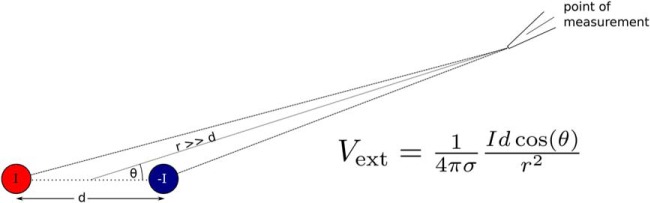
Dipole model consisting of a current sink (red) and a current source (blue) separated by *d*. The point of measurement represents a possible recording location where extracellular potential, *V*_ext_, is recorded. For the far-field approximation to hold the distance from the dipole *r* should be much larger than the distance between the sink and source (*d*). See text for more detail. *I* is current intensity, σ is extracellular medium conductivity, and θ is the angle measured from the dipole axis.

In conclusion, we found that for horizontal, and not vertical, recordings the peak-to-peak amplitude decayed with the inverse square of the distance from the soma (k≈−2; Fig. 8*B*). This inverse-square law is theoretically predicted at distances to the dipole much greater than the separation between the current source and sink (i.e., a far-field approximation; Fig. 9; Nunez and Cutillo, 1995; Griffiths, 1999). Note also that the profile of the potential obtained in detailed morphology models did not agree with this prediction. As discussed above, the potential in these models does not decrease uniformly with the distance from soma (compare Fig. 8*B*,*C*, insets, Fig. 5*B*), but can abruptly increase. These increases are most likely due to the passage of the measurement line through an area occupied by a dendrite (Fig. 5*A*,*B*, arrows).

### Peak-to-peak amplitude of EAP weakly depends on the soma–AIS distance

We next investigated whether the soma–AIS distance can influence the peak-to-peak amplitude of the EAP. The peak-to-peak amplitude of the far-field dipole potential measured at a fixed position depends on the product between the dipole current (*I*, the axial current between soma and AIS) and separation between the poles (*d*, the soma–AIS distance; Fig. 9). Therefore, increasing the distance of the AIS from soma might increase the peak-to-peak amplitude of the EAP, but numerical simulations of the simplified soma–AIS model showed only a weak dependence of the peak-to-peak amplitude on the soma–AIS distance (Fig. 8).

**Figure 10.**
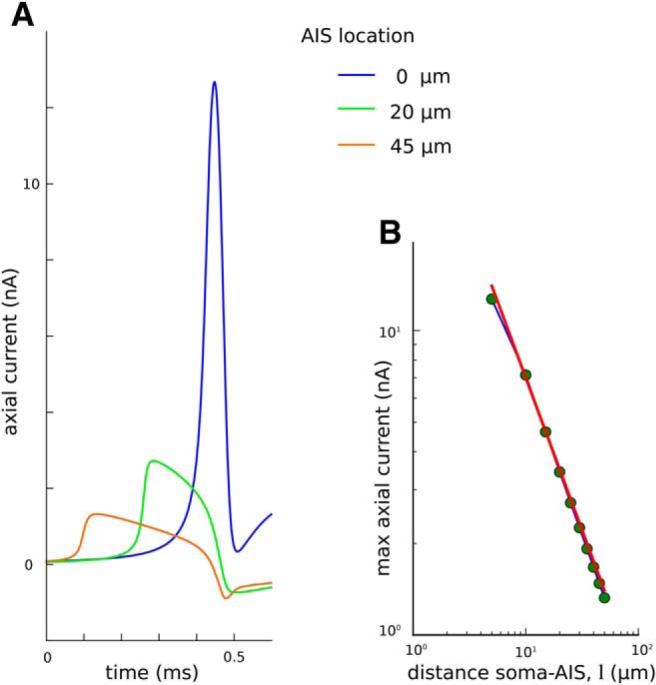
Dependence of axial current peak-to-peak amplitude on the soma–AIS distance in the soma–axon model. ***A***, Axial current passing from the axon to the soma during the action potential, aligned to the peak of somatic AP (which is at 0.5 ms). ***B***, The maximum of the axial current vs the distance of the AIS end proximal to the soma in double-logarithmic scale. The red line shows the fitted function $I_{axial}=(70\;\text{nA}\cdot \mu \text{m})/l$ (which is a linear function in double-logarithmic scale).

To explain this finding, we investigated the effect of the soma–AIS distance on the axial current generated during the action potential. We found that the peak-to-peak amplitude of the axial current decreased with the inverse of the soma–AIS distance, *l* (Fig. 10*A*). Indeed, we found that it was possible to fit a straight line of slope *a* = −1 through the points representing the logarithm of the maximum axial current versus the logarithm of the soma–AIS distance (Fig. 10*B*). This linear relation confirms that the peak-to-peak amplitude of the axial current is inversely proportional to the distance between the soma and the AIS, $I_{axial}∼1/l$. Such a relationship is also predicted by the resistive coupling hypothesis (Hamada et al., 2016). This drop of current magnitude compensates for the increase between the sink and source of the dipole (soma and AIS). Since the product of current intensity, *I*, and the dipole dimension, *d* (which is approximately equal to *l*), remains constant, the peak-to-peak amplitude depends only weakly on the soma–AIS distance.

### EAP broadens with soma–AIS distance

**Figure 11.**
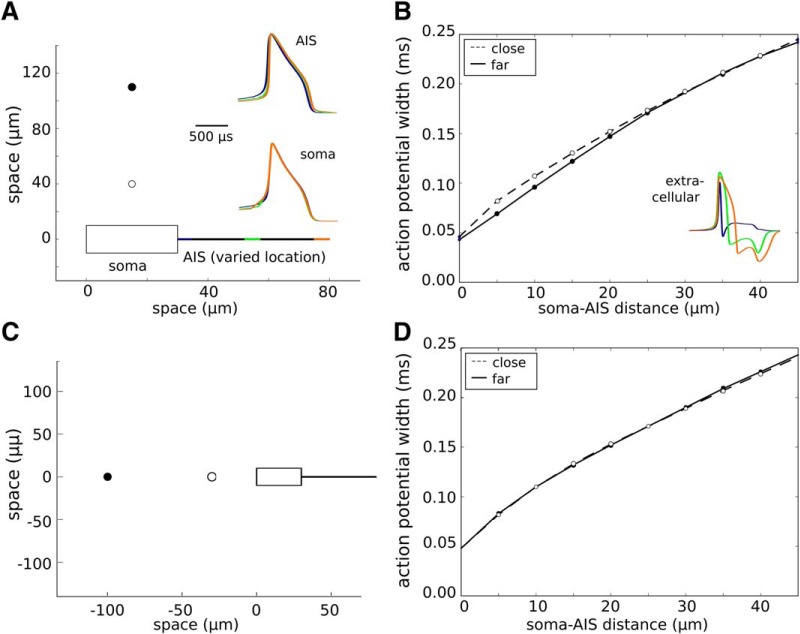
Width of the EAP as a function of the soma–AIS distance. ***A***, Schematic representation of the soma–axon model (bottom) and their relation to the recording points (dots above soma). The soma–AIS distance was systematically varied from 0 (directly attached to the soma) to 45 μm. Insets, Waveforms of action potentials recorded intracellularly in the AIS (top inset) and in the soma (bottom). The waveforms are normalized to the peak of the somatic potential. ***B***, Action potential width measured at half peak-to-peak amplitude as a function of the soma–AIS distance for two different recording locations (close, 30 μm above the soma; far, 100 μm above the soma). Inset, Examples of EAP waveshapes for three different locations of AIS (recorded 40 μm above the soma). ***C***, Schematic representation of the soma–axon model (right) and the relation to the recording points (dots left to the soma). ***D***, Action potential width measured at half peak-to-peak amplitude as a function of the soma–AIS distance for the two different recording locations (close, 30 μm left of the soma; far, 100 μm left of the soma).

To study the effect of the soma–AIS distance on the EAP width, we calculated the extracellular potentials generated by models with the AIS placed at 10 different distances from the end of the soma, up to 45 μm distally. We observed that the EAPs become gradually wider with increasing distance between the soma and the AIS (Fig. 11*B*), while the shapes of intracellular waveforms remain similar (Fig. 11*A*, insets). The functional form of this dependence changes only slightly with the location of the recording site: measurements in two perpendicular directions from soma (Fig. 11, compare *B*, *D*) give the same dependence on the soma–AIS distance.

## Discussion

Using detailed morphologic models of reconstructed neurons and simplified soma–axon models, we have shown that extracellular action potentials can be reconstructed from the current dipole formed by the soma and AIS at their initiation. We also show that the EAP shape depends on the position of the recording electrode with respect to the neuron promoting the extracellular contribution of different compartments of the neuron. In addition, while the width of the EAPs varies with the soma–AIS distance, their peak-to-peak amplitudes remain relatively constant.

The contribution of the AP to the extracellular field is shaped by the structure of the dendritic tree and the site of AP initiation. A large body of experimental data supports the more distal initiation in the axon initial segment (Palmer and Stuart, 2006), but the impact of axonal initiation on the EAP had not been examined before. Using simplified models, we showed that in the initial phase of the AP, the soma and AIS form a current dipole, whose contribution to the extracellular potentials decays inversely with the square of the distance from the dipole. At large distances (far-field approximation), the dipole contribution to the extracellular field does not depend on the separation between the AIS and the soma. In contrast, the width of the EAP increases with the soma–AIS distance. This soma–AIS dipole is different from the soma–dendrites dipole known from standard models (Gold et al., 2006). In fact, we showed that reducing the density of sodium channels in the AIS shifts AP initiation to the soma, and, as a consequence, that the extracellular potential is dominated by the soma–dendrites contribution.

Our results provide an important insight into the understanding of EAPs. It is known that the shape and the peak-to-peak amplitude of the EAPs vary depending on the location of the recordings (Gold et al., 2006). Also, different types of neurons display EAPs of different widths, such as excitatory cells, which tend to have broader EAPs when compared with interneurons (McCormick et al., 1985; Barthó et al., 2004), although there are exceptions (Vigneswaran et al., 2011). To separate the action potentials of multiple neurons recorded extracellularly, it is common to use the waveform features of an EAP, such as the half-widths of the positive and negative peaks, the interval between them, and the difference in their peak-to-peak amplitudes (Lewicki, 1998; Einevoll et al., 2012). In addition, these and other waveform features sometimes allow the identification of neurons of different types (Peyrache et al., 2012; Dehghani et al., 2016). However, the significance of such features and their biophysical underpinnings is not completely understood. Numerical simulations of the extracellular potentials around reconstructed morphology of CA1 pyramidal neurons showed that the width of the EAP increases proportionally with the distance between the soma and the recording electrode (Gold et al., 2006). In addition, in this study the shape and peak-to-peak amplitude of the EAP was strongly affected by the channel densities in the dendrites and in the axon initial segment. In our work, we show that the extracellular features of action potentials also depend on the exact location of their initiation site.

In this article, we analyzed the EAP of a pyramidal neuron, but critical resistive coupling theory shows that there is no qualitative difference in the mechanisms of the AP generation, as long as the AIS is connected to a big soma and/or extensive dendritic tree (relative to axon diameter). In this case, the somatic and dendritic compartments act as a current sink clamping the AIS potential and leading to sharp AP initiation (Telenczuk et al., 2017). Since smaller neurons, such as basket cells, have smaller axons, critical-resistive coupling theory should still hold and the extracellular signature of spikes would be qualitatively similar. However, there could be quantitative differences. If the axial current matches the neuron size as hypothesized in the study by Hamada et al. (2016), the peak-to-peak amplitude of the extracellular spike should grow with the size of the neuron. The duration or shape will depend on the type and densities of ion channels. One possibility is that the AP is not regenerated at the soma, as for example in chick auditory brainstem (Kuba et al., 2006), so the shape will be different although the dipolar distribution of currents in soma and AIS still holds (Kole and Brette, 2018).

Finally, our results show that it should be possible, and of great interest, to follow experimentally the dynamic change of the soma–AIS distance by means of extracellular recordings. The length of AIS and soma–AIS distance vary between neurons of the same and different types (Kuba et al., 2006; Fried et al., 2009). Furthermore, the AIS is plastic and its length and distance from the soma can change as a result of elevated activity, which could occur due to plastic changes in a timescale of hours (Evans et al., 2015) to days (Grubb and Burrone, 2010; Evans et al., 2013; Muir and Kittler, 2014). This also happens as a consequence of a disease such as a stroke (Schafer et al., 2009; Hinman et al., 2013). Therefore, we expect that the shape of the EAP will vary according to the soma–AIS distance, such that long-term recordings from the same neuron could show gradual increase of the AP width. Since, the plasticity of AIS was never studied *in vivo* from intact neurons, this may open new methods of visualizing such dynamic changes and investigating their functional role.

Our results are consistent with the large variability of EAP waveforms recorded *in vivo* (Fee et al., 1996; Harris et al., 2000). It is known that the waveshapes of the EAP depend on the position of the electrode, the morphology of the neuron, and the densities of ion channels (Henze et al., 2000; Barthó et al., 2004; Gold et al., 2007; Pettersen and Einevoll, 2008). In particular, the presence of positive initial peak, as observed in our model, has been recognized in some studies (Palmer and Stuart, 2006). To further test our model experimentally, one could record the extracellular potentials *in vitro* at multiple sites using multishank electrodes coregistered with the soma–AIS distance. The AIS can be localized using fluorescent sodium channel markers (e.g., CoroNa) or immunostaining (e.g., anykrin G is specific to AIS and the nodes of Ranvier; Zhou et al., 1998). This setup might allow for testing the following two new predictions of the model: (1) the presence of a positive peak at the beginning of the EAP in the vicinity of soma–AIS region; and (2) the width of the EAP as a function of the soma–AIS distance. In the latter case, we would need to visualize the change of soma–AIS distance dynamically, probably over the course of many hours or days (Grubb et al., 2011). Such recordings are technically challenging, but are possible using present technology (Grubb and Burrone, 2010).

At the population level, the contribution of neurons to the LFP depends critically on the presence of voltage-dependent channels and neuronal morphology. For example, during the up-state, the LFP contains larger contributions from the active potassium and sodium currents than from synaptic currents (Reimann et al., 2013); similarly, active conductances in the dendrites were shown to have a major impact on the spectrum of the field potential (Ness et al., 2016). The structure of the dendritic tree has also been implicated in the generation of LFP signals (Lindén et al., 2010). Results in the present work suggest that the biophysics of the axon and the site of the action potential initiation may be additional factors determining the peak-to-peak amplitude and the extracellular potentials. The effects of the soma–AIS distance on the LFP generated from a network of multicompartmental model neurons is an interesting outlook of the present work.

Acknowledgments: We thank Jennifer Goldman and Fabrizio Gabbiani for their comments on the manuscript.

10.1523/ENEURO.0068-18.2018.supplement Extended Data Supplementary software. Download Extended Data, ZIP file.

## Synthesis

Reviewing Editor: Leonard Maler, University of Ottawa Decisions are customarily a result of the Reviewing Editor and the peer reviewers coming together and discussing their recommendations until a consensus is reached. When revisions are invited, a fact-based synthesis statement explaining their decision and outlining what is needed to prepare a revision will be listed below. The following reviewer(s) agreed to reveal their identity: James Jun, Torbjørn Ness

Editorial comments.

Both reviewers agree that this manuscript is well done and advances the field by providing a better mechanistic understanding of the extracellular action potential. This understanding will have wide reaching implications for neuroscience research that utilizes extracellular recordings from many neurons and then applies automated spike sorting algorithms to extract individual spike trains. As the recording methodology improves, the spike sorting algorithms will need to keep pace and this Ms may help to provide a principled basis for such algorithms. Both reviewers required changes to the Ms but it will not required re-review. I have included the important reviewer comments below.

The authors should provide an detailed response to all the points raised (rebuttal) and also provide a copy of the Ms with the changes highlighted.

This manuscript examines the role of axon initial segment (AIS) in generating the extracellular action potential (EAP) and its spatiotemporal properties. To demonstrate this, the authors used a layer 5 neuron model (Hallermann'12) with realistic morphology and sodium channel distribution, and a simplified soma-axon model. 

Although these are solid evidences, it would be helpful to convince wider audiences to include multiple cell types such as GABAergic interneurons (basket cells) and Layer 2/3 cells and relate to the simplified soma-axon model by varying the soma-AIS distance and other relevant parameters. 

EDITOR NOTE: I would not require a complete model of other cell types but I would like, at the least, and indication of how such models might be provided. I think this would be important in terms of the use of this Ms for spike sorting algorithms.

[Specific comments] 

Figs 4,5,6: I agree that comparison of the absolute amplitudes are not meaningful (Line 99). I suggest changing the color scales to the normalized amplitude scale, which is currently expressed in microvolts. 

Figure 8: axis labels for panels B and C are inconsistent.

Advances

Modelling of extracellular potentials is gaining in popularity, and the availability of biophysically detailed cell models is steadily increasing. However, such models often do not explicitly model the axon initial segment, and the impact this has on calculated extracellular action potentials (EAPs) has been unclear. Many modellers of EAPs are not even aware that this is an important issue. This manuscript provides a basic understanding of this issue, and can therefore be an important contribution to the modelling of extracellular potentials.

Software comment

The source code of reproducing the figures was provided, but perhaps the README file could have been improved (e.g., add folder to .bashrc file, compile .mod files and so on). It could probably quite easily be made compatible with python3 as well, but I understand that this might not be prioritized.

In general I think the manuscript is good, but I have a few comments: 

1) It is somewhat unclear if the description of the model given in L84-L88 is modified from the original model, or just a restatement of the original model. 

2) L102-105: The AP is initiated by current injection, which forms a monopole that later has to be subtracted. It should be relatively easy to do the current injection in NEURON using a POINT_PROCESS instead of an ELECTRODE_CURRENT, and this would remove the monopole without the need for the subtraction afterwards. Alternatively, the AP could be evoked by a synaptic input. Perhaps the authors could verify that their results are valid also under either of these conditions? 

3) Figure 8 could be improved a bit. As far as I understand it, B and C shows basically the same data, but the axes are labeled different. Perhaps the authors could also add the information from Figure 5Ba (the part above the soma) in the same plot for reference, to illustrate the discussion in L241-244. Perhaps it could also be made more clear what is meant by the sentence at L245. 

4) L267-269: “Since the product of current intensity, I, and the dipole dimension, d, remains constant, the EAP amplitude does not depend on the AIS position.” 

I think this statement is too strong. The EAP amplitude will at some positions definitely be dependent on the position of the AIS, for example, in the immediate vicinity of the AIS. Perhaps the statement should rather be that the EAP amplitude is only weakly dependent on the distance between the soma and the AIS? 

5) In Figure 11, the recording positions are perpendicular to the axis of the axon, while in Figure 8, the recording positions are along the axis of the axon. Is there any particular reason for this difference? 

6) There are some inconsistencies in the language. The amplitude of the EAP is called peak-to-peak amplitude, peak to peak amplitude, peak to valley, field amplitude, voltage, electric field, extracellular potential etc. In my experience ‘electric field’ is used to denote the vector field, \vec E, while this paper concerns the gradient of this field. These inconsistencies are especially clear in the figure axis labels. The x-axis of Fig. 11B is labeled ‘AIS position’, while the same feature is labeled ‘distance soma-AIS, l’ in Figure 10B, and there are many more examples.
